# Comparison of students’ perceptions of online and hybrid learning modalities during the covid-19 pandemic: The case of the University of Sharjah

**DOI:** 10.1371/journal.pone.0283513

**Published:** 2023-03-28

**Authors:** Tareq M. Osaili, Leila Cheikh Ismail, Hussein M. ElMehdi, Anas A. Al-Nabulsi, Asma’ O. Taybeh, Sheima T. Saleh, Hanin Kassem, Hana Alkhalidy, Habiba I. Ali, Ayesha S. Al Dhaheri, Lily Stojanovska

**Affiliations:** 1 Department of Clinical Nutrition and Dietetics, University of Sharjah, Sharjah, United Arab Emirates; 2 Department of Nutrition and Food Technology, Jordan University of Science and Technology, Irbid, Jordan; 3 Nuffield Department of Women’s & Reproductive Health, University of Oxford, Oxford, United Kingdom; 4 Department of Applied Physics and Astronomy, University of Sharjah, Sharjah, United Arab Emirates; 5 Department of Nutrition and Health, College of Medicine and Health Sciences, United Arab Emirates University, Al Ain, United Arab Emirates; 6 Institute for Health and Sport, Victoria University, Melbourne, Australia; Presbyterian University College, GHANA

## Abstract

Hybrid learning enables educators to incorporate elements of conventional face-to-face learning methods with structured online schemes. This study aimed to assess university students’ perceptions of online and hybrid learning during the ongoing COVID-19 pandemic. A web-based cross-sectional study was conducted at the University of Sharjah, in the United Arab Emirates (n = 2056). Students’ sociodemographic characteristics, perceptions of online and hybrid learning, concerns, and university life changes, were investigated. Perception statements were dichotomized into "positive" and "negative" based on a 50% cut-off point. Scores of > 7 and >5 indicated positive perceptions of online and hybrid learning respectively while scores of ≤ 7 and ≤ 5 indicated negative perceptions. Binary logistic regression analysis was performed to predict students’ perceptions of online and hybrid learning according to demographic variables. Spearman’s rank-order correlation was performed to determine the relationship between students’ perceptions and behaviors. Most students preferred online learning (38.2%) and on-campus learning (36.7%) to hybrid learning (25.1%). Around two-thirds of the students had a positive perception of online and hybrid learning in terms of university support, however, half of them preferred the assessment during online or on-campus learning. Main difficulties reported in hybrid learning were lack of motivation (60.6%), discomfort when on-campus (67.2%), and distraction due to mixed methods (52.3%). Older students (p = 0.046), men (p<0.001), and married students (p = 0.001) were more likely to have a positive perception of online learning, while sophomore students were more likely to have a positive perception of hybrid learning (p = 0.001). In this study, most students preferred online or on-campus over hybrid learning and expressed certain difficulties while on hybrid learning. Future research should focus on investigating the knowledge and capability of graduates from a hybrid/online model compared to a traditional model. Obstacles and concerns should be considered for future planning to ensure the resilience of the educational system.

## Introduction

The coronavirus disease (COVID-19) has rapidly spread worldwide, forcing countries to apply strict social distancing measures and lockdowns affecting people’s lives ever since its emergence in late 2019 [1]. Similar to most sectors within communities, the COVID-19 pandemic has greatly impacted the education process at all levels of pedagogical institutions [2]. Hence, numerous governments globally, including the UAE have made prompt decisions to hinder the spread of COVID-19 and ensure safety for all, by avoiding face-to-face interaction among students and between students and instructors [3]. The United Nations Educational, Scientific and Cultural Organization (UNESCO) estimated that more than 186 countries prevented face-to-face learning affecting more than 1.3 billion students globally and 1.3 million learners in the UAE [4]. As a result, remote learning was implemented in public and private schools as well as higher education institutions in the UAE. On-campus teaching was suspended and classes shifted from the classical classroom environment to virtual platforms using the relevant online communication platform technologies [5]. Therefore, the new normal amid the COVID-19 pandemic involved a swift shift from traditional to online classes, personal to virtual teaching, and seminars to webinars [6].

Literature shows that students’ learning experiences whilst online learning can be as successful as traditional methods and have been shown to result in more satisfaction among students and higher attainment of information [7–9]. Online learning offers the benefit of flexibility that traditional learning may not always offer, as well as wider communication and collaboration when using web software and online video meetings increasing social interaction [10]. A study among university students in the UAE revealed that students believe that they would perform well after shifting to online learning amid the pandemic [11]. Given the unprecedented COVID-19 pandemic, the sudden shift to remote learning is ought to vary greatly from planned online learning that schools or higher education institutes may usually implement. Despite this shift being highly effective in containing the spread of the virus amongst students, many factors are suggested to have affected the learning and teaching experience. Some challenges and obstacles can be related to connectivity and technical issues, socioeconomic status, inadequate training and experience, work and information overload, and challenges concerning courses requiring special equipment or laboratory research [12–15]. Further research shows that whilst on remote learning during the pandemic, students are less attentive, and less motivated compared to before the pandemic [16].

As vaccination rates increased and the number of COVID-19 confirmed cases stabilized, suggestions for returning to in-person teaching have emerged [17]. Lessons learned from following an online learning mode during the pandemic suggested merging certain aspects and features of online learning with face-to-face learning mode [9]. In the broad context, blended learning, or hybrid learning as it will be referred to in this study, is a method that enables educators to incorporate elements of conventional face-to-face learning methods with structured online schemes and has been implemented previously in tertiary education for multiple purposes [18, 19]. It is regarded as an effective learning style in terms of student outcomes, enabling more flexibility and access to materials, allowing the convenience of studying at one’s own pace, and providing prompt feedback [20]. Before the COVID-19 pandemic, the usefulness and impact of hybrid learning methods on student learning outcomes, preference, and satisfaction have been recorded in multiple studies. In research concerning health-related programs, hybrid learning has been proven to be similar to or more effective than traditional learning in performance assessment and evaluation [18, 21]. Other research among university students has shown that the students were in favor of the hybrid learning method as it provided them with a more flexible learning environment [22, 23]. Further, this method has been shown to have a positive impact on encouraging innovative and supple learning outcomes [24]. As higher education institutions are adopting hybrid systems, challenges entail ensuring the provision of a high-quality learning experience for both settings.

To the best of our knowledge, very limited research has explored students’ perceptions of online learning and hybrid learning systems during the COVID-19 pandemic. Since the students are accustomed to the traditional learning approach; an on-site pattern, it was expected that they would not keenly prefer online/ hybrid learning. At the University of Sharjah, the hybrid learning system was implemented as the COVID-19 status stabilizes in the fall semester of 2021/2022 taking into consideration the possibility of a new wave of COVID-19 outbreak and being well prepared to carry out online or the hybrid learning mode [12]. Therefore, this study aims to assess student perceptions of the online and hybrid learning modes among the university of Sharjah students post its implementation during the COVID-19 pandemic.

## Methods

### Study design and participants

This was a cross-sectional study conducted between November 2021 and January 2022 among university students who were enrolled at the University of Sharjah at the time of data collection. The minimum required sample size was calculated based on the following equation with a confidence interval of 99%:

$$N=z^{2}\times P\times (1-P)/e^{2}$$

Where z = 2.576; P = (estimated proportion of the population that presents the characteristic) = 0.5; e (margin of error) = 0.05; N (sample size) = 664 participants, plus 20% (attrition rate) = approximately 797 participants.

Students were encouraged to participate in the study through a web link connecting to the online survey which was shared with them through the university’s online platform. A total of 2056 participants were interested in the study and filled out the survey questionnaire. An information sheet explaining the objective of the study, its significance, and the protocol of the study was provided on the first page followed by a consent form. Written informed consent was obtained electronically from all participants before completing the survey. Only consenting participants were then directed to complete the online questionnaire. Participation was completely voluntary, and the students were free to exit the online survey at any point before submission. No personal identification information was collected, and participation was completely anonymous. The study protocol obtained ethical approval from the Research Ethics Committee at the University of Sharjah (Reference number: REC-21-10-20).

### Survey questionnaire

A self-administered, multi-component questionnaire was developed to investigate the students’ perceptions of online and hybrid learning. A first draft of the survey questions was prepared by researchers at the University of Sharjah based on a thorough review of available relevant literature [7–9]. The survey questionnaire was first prepared in English, translated to Arabic, and back-translated to English to ensure consistency between the two versions. Then the survey was pilot tested on a group of 30 students to ensure that the survey is feasible and understandable. The pilot testing data was not included in the results. The results of the Cronbach alpha test indicated a good internal consistency level with a value ranging between 0.735 and 0.873.

The online questionnaire included 41 items and consisted of four sections. The first section included sociodemographic questions inquiring about age, sex, marital status, nationality, the emirate of residence, level of university education, academic year enrollment, COVID-19 vaccination status, and if a previous positive test result of COVID-19 was obtained. An additional question inquired about the student’s preferred mode of studying (response options; Online, Hybrid, or On-Campus). The second section focused on students’ perceptions of the online and hybrid learning modes. Students were asked to provide their opinion about online learning (15 items) and the hybrid learning mode (11 items) using a 3-point Likert scale (response options; Agree, Neutral, Disagree). The statements enquired about students’ perceptions of their overall experience with the learning modes, their understanding of materials taught, the engagement and communication with instructors, the support provided by the university and examinations. The following section comprised five items focused on students’ concerns, expected difficulties as well as preparations for returning to campus. As the study was conducted two months into the implementation of the hybrid learning mode, the last section included four items that focused on changes in students’ university life and the use of university facilities. The questionnaire took approximately 10 to 15 minutes to complete.

### Statistical analysis

Categorical variables were reported as counts and percentages and continuous variables as means and standard deviations (SD). Cross tabulations and chi-square tests were used to compare indicators across demographic characteristics. Students’ levels of agreement on a range of perception statements related to online (15 items) and hybrid learning (11 items) were explored using a three-point Likert scale and were dichotomized into "positive" and "negative" based on a 50% cut-off point, in line with several previous studies [25, 26]. For online learning perceptions, scores of > 7 were considered positive, and scores ≤ 7 were considered negative. While, for the hybrid learning perception, scores of > 5 indicated positive perceptions, and scores ≤ 5 indicated negative ones. A Spearman’s rank-order correlation was performed to determine the relationship between students’ perceptions and behaviors. It should be noted that the analysis was focused on hybrid learning only, as this learning modality allowed for some degree of on-campus activity compared to online learning. Results were significant at p-value < 0·05. Statistical analysis was performed using Statistical Package for the Social Sciences (SPSS) ver. 26·0 (IBM, Chicago, IL, USA).

## Results and discussion

### Participants’ profile

A total of 2056 students participated in the study. Table 1 represents the socio-demographic characteristics of the respondents. More men than women completed the online survey with a ratio of 3:1 (76.0% and 24.0% respectively). Majority of the respondents were single (84.6%), national citizens (62.8%), and enrolled in undergraduate programs (92.0%). Around half of the respondents were 18–20 years and a third of them were between 21–26 years old. Most of our respondents were Sophomores (2^nd^ and 3^rd^ year) (52.7%) or Freshmen (1^st^ year) (33.0%), while the least proportion were Seniors (4^th^ and 5^th^ year) (14.3%). Concerning the questions about COVID-19, most of the respondents reported being vaccinated (93.5%), and around a third of all participants reported being previously infected with COVID-19 (27.1%).

**Table 1:** Demographic characteristics of participants and preferred mode of learning by demographic factors (n = 2056).

Variables	n(%)	Online n(%)	Hybrid n(%)	On-campus n(%)	p-value
**Total**		785 (38.2)	517 (25.1)	754 (36.7)	
**Sex**					
Women	493 (24.0)	134 (17.1)	138 (26.7)	221 (29.3)	p<0.001*
Men	1563 (76.0)	651 (82.9)	379 (73.3)	533 (70.7)	
**Age (years)**					
18–20	1100 (53.5)	229 (29.2)	321 (62.1)	550 (72.9)	p<0.001*
21–26	683 (33.2)	341 (43.4)	158 (30.6)	184 (24.4)	
More than 26	273 (13.3)	215 (24.4)	38 (7.4)	20 (2.7)	
**Marital status**					
Single	1739 (84.6)	534 (68.0)	474 (91.7)	731 (96.9)	p<0.001*
Married	295 (14.3)	235 (29.9)	40 (7.7)	20 (2.7)	
Divorced/widowed	22 (1.1)	16 (2.0)	3 (0.6)	3 (0.4)	
**Nationality**					
UAE National	1291(62.8)	623 (79.4)	287 (55.5)	381 (50.5)	p<0.001*
Resident Expatriate	765 (37.2)	162 (20.6)	230 (44.5)	373 (49.5)	
**Current educational level**					
Undergraduate	1891 (92.0)	695 (88.5)	466 (90.1)	730 (96.8)	p<0.001*
Postgraduate	165 (8.0)	90 (11.5)	51 (9.9)	24 (3.2)	
**College level**					
Freshmen	678 (33.0)	198 (25.2)	186 (36.0.)	294 (39.0)	p<0.001*
Sophomore	1083 (52.7)	466 (59.4)	252 (48.7)	365 (48.4)	
Senior	295 (14.3)	121 (15.4)	79 (15.3)	95 (12.6)	
**COVID-19 vaccination status**					
Vaccinated	1923 (93.5)	714 (91.0)	486 (94.0)	723 (95.9)	p<0.001*
Unvaccinated	133 (6.5)	71 (9.0)	31 (6.0)	31 (4.1)	
**Previous COVID-19 infection**					
Yes	558 (27.1)	234 (29.8)	130 (25.1)	194 (25.7)	p = 0.099
No	1498 (72.9)	551 (70.2)	387 (74.9)	560 (74.3)	

* P-values for chi-square tests were calculated among demographic categories p-value <0.05 was considered to be statistically significant

The implementation of hybrid learning at the University of Sharjah during the COVID-19 pandemic facilitated transitioning from full online learning while ensuring student and staff safety [12]. Regarding the preferred mode of learning, an almost equal proportion of students preferred either online learning or on-campus traditional learning (38.2% and 36.7% respectively), while hybrid learning was preferred by a lesser proportion of students (25.1%). In comparison, in a study among Albanian students, about half of the participants reported adapting easily to online learning during the COVID-19 pandemic [15]. However, in another study in Jordan, most students were not in favor of the online learning approach and thought that it negatively impacted the quality of learning [13].

Results of the chi-square test showed that there were statistically significant associations between variables (age, gender, marital status, nationality, educational level and studying year, and vaccination status) and preferred learning mode (p<0.001). According to the results, women preferred the on-campus learning style more than men, who preferred the online learning style. These findings could be due to the sociable nature of women as they are usually more socially oriented than men [27] and previous research shows that men have more confidence in using technology for learning purposes than women [28]. However, this was different from the students in Jordan where women were found to be more optimistic and in favor of online learning [13]. Students between 18 to 20 years old, preferred on-campus learning more than students of other age groups. Moreover, it was observed that freshman students preferred the on-campus learning mode more than sophomore and senior students, who preferred online learning more than other types. Undergraduate students had a higher preference for on-campus learning than postgraduate students who preferred online learning. Older students may perceive online classes as more convenient in case of working, or having a more demanding lifestyle, whereas younger students may have the enthusiasm to live the traditional campus life. Preference for online learning was higher among unvaccinated students, whereas vaccinated students preferred on-campus learning (p = 0.002). Further, those who had a previous COVID-19 infection preferred online learning while their counterparts had a higher preference for on-campus and hybrid learning (p = 0.099).

### Perceptions of hybrid and online learning

Amid the COVID-19 pandemic, a large emphasis was put on universities’ provision of sufficient technical support to motivate students in the emergency learning process and manage any technical stresses that may arise [29]. Students’ levels of agreement on a range of perception statements related to online and hybrid learning were explored and presented in Table 2. Around two-thirds of the students agreed that they got used to the online learning experience (62.9%). Moreover, two-thirds of the students agreed that the university provided them with the needed technical support (65.9%) while a lesser proportion agreed that they were provided with the needed personal support (54.4%). This was similar to students in Malaysia [14] but in contrast to studies in different countries such as Romania [30]. Students’ positive perceptions in the present study could be attributed to the fact that online learning and blended methods have been long integrated at the University of Sharjah, UAE before the COVID-19 pandemic. This could have favorably provided a basis for the experience required to smooth the transition to remote learning amid the pandemic [12]. Concerning student-instructor interaction, most of the students found it easy to communicate (62.7%), ask questions (64.4%), and engage with the instructor during online classes (56.4%) and that the instructor was available during office hours (76.9%). This was a favorable result, as in an exploratory study among educators in the UAE, only one in five educators believed that online classes can promote student engagement [31].

**Table 2:** Students’ perceptions of online and hybrid learning (n = 2056).

	Disagree	Neutral	Agree
	N (%)		
**Perceptions of online learning**			
I got used to the online learning experience	385 (18.7)	344 (16.7)	1327 (64.5)
Explanations and lecturing in theoretical lectures are better in Distance learning	687 (33.4)	293 (14.3)	1076 (52.3)
Practical sessions are better in Distance Learning	911 (44.3)	264 (12.8)	881 (42.9)
It was easy for me to engage with the Professor in Distance Learning	653 (31.8)	244 (11.9)	1159 (56.4)
It was easy for me to ask questions during Distance Learning lectures	504 (24.5)	227 (11.0)	1325 (64.4)
It was easy for me to communicate with my instructor during Distance Learning	513 (25.0)	253 (12.3)	1290 (62.7)
The instructor was available during office hours	158 (7.7)	317 (15.4)	1581 (76.9)
The University provided you with the needed support (technically)	333 (16.2)	369 (17.9)	1354 (65.9)
The University provided you with the needed support (personally)	372 (18.1)	566 (27.5)	1118 (54.4)
Online Exams are fair	547 (26.6)	267 (13.0)	1242 (60.4)
Grading in online exams was fair and better than in-person exams	608 (29.6)	393 (19.1)	1055 (51.3)
Proctoring online exams was NOT effective	1260 (61.3)	350 (17.0)	446 (21.7)
It was easy to cheat in online exams	1404 (68.3)	323 (15.7)	329 (16.0)
Questions in Online Exams were easy to read and understand	406 (19.7)	357 (17.4)	1293 (62.9)
The online exam format was better than the in-person exams	491 (23.9)	385 (18.7)	1180 (57.4)
**Perceptions of hybrid learning**			
Hybrid learning system could be distracting due to mixed learning methods and approaches	476 (23.1)	505 (24.6)	1075 (52.3)
I feel unmotivated to be in a hybrid learning system	674 (32.8)	611 (29.7)	771 (37.5)
The university provides students with excellent technical support	319 (15.5)	337 (16.4)	1400 (68.1)
Instructions and guidelines are provided regularly	165 (8.0)	277 (13.5)	1614 (78.5)
The university provides us with excellent online support services	266 (12.9)	277 (13.5)	1513 (73.6)
The university provides us with excellent personal support	411 (20.0)	434 (21.1)	1211 (58.9)
The university provides us with financial aid and support	602 (29.3)	608 (29.6)	846 (41.1)
I achieve better in hybrid classes compared to online classes	549 (26.7)	588 (28.6)	919 (44.7)
I can manage my time better in hybrid classes compared to online classes	724 (35.2)	472 (23.0)	860 (41.8)
I feel comfortable when I visit the university campus during hybrid learning mod	1246 (60.6)	0 (0.0)	810 (39.4)
I understand the course material better through on-campus learning	642 (31.2)	0 (0.0)	1414 (68.8)

In the present study, around half of the students found that the theoretical lectures are better in online classes while a lesser proportion agreed that the practical sessions are better in distance learning (52.3% and 42.9% respectively). Regarding the examinations during online learning, around 60% of the students agreed that the questions were easy to understand, the exams are fair, and the exam format was better than in-person exams, while around half of them (51.3%) agreed that grading was fair and better than in-person exams. This was similar to a previous study in the UAE, where most of the students agreed that online assessment was easy to understand and follow and that they achieved better grades in online examinations [32]. On the other hand, 68.3% disagreed that it was easy to cheat in online exams and 61.3% disagreed that proctoring online exams was not effective.

Regarding students’ perceptions of hybrid learning, most of the students agreed that instructions and guidelines are provided on regular basis (78.5%). Moreover, around two-thirds agreed that the university provides students with excellent online support services, technical support, and excellent personal support (73.6%, 68.1%, and 58.9% respectively)., More than two-thirds agreed they understand the course material better through on-campus learning (68.8%). However, less than half of the students agreed that they achieved better in hybrid classes than in online classes (44.7%). On the other hand, around a third of the students feel comfortable when visiting the university campus and are motivated during hybrid learning mode (39.4% and 32.8% respectively). About two of five students feel that they were able to manage their time better in hybrid classes than in online ones (41.8%). Furthermore, about half of the students felt that hybrid learning could be distracting due to the mixed learning methods (52.3%).

The average score for students’ perceptions of online learning was 9.35 (out of 15) with a score percentage of 62.3% based on the criteria mentioned earlier. As for the perceptions of hybrid learning, the average score was 6.59 (out of 11) with a score percentage of 60.0%. The detailed distribution of students’ perception categorization is available in the supplementary material (S1 Table). A binary logistic regression analysis was performed to understand whether students’ perception of online and hybrid learning can be predicted based on different demographic variables (the dependent variable is "perception scores", measured on a dichotomous scale–"positive" or "negative"–and the independent variables: "demographics") as shown in Table 3. Logistic regression results indicated that students in the group age (>26 years) are 2.9 times more likely to have positive perceptions of online learning (OR: 2.950, 95% CI: 1.768–4.921). Men (OR: 1.256, 95% CI: 1.004–1.572) and married (OR: 2.303, 95% CI: 1.417–3.744) students are 1.25 and 2.3 times more likely to have positive perceptions of online learning, respectively. Also, the likelihood of having a positive perception of online learning was higher among non-Emirati students (OR: 0.423, 95% CI: 0.346–0.517). However, regarding the hybrid learning mode, being a sophomore student (OR: 0.673, 95% CI: 0.536–0.845) was significantly associated with higher odds of having positive perceptions of hybrid learning.

**Table 3:** Predictors of students’ perceptions of online and hybrid learning using logistic regression analysis.

Items	Online learning		Hybrid learning	
	OR (CI)	p-value	OR (CI)	p-value
**Gender**		p = 0.046*		p = 0.240
Women	Reference		Reference	
Men	1.256 (1.004–1.572)		0.871 (0.691–1.097)	
**Age**				
18–20	Reference	p = 0.000*	Reference	p = 0.585
21–26	2.226 (1.713–2.894)	p = 0.000*	0.877 (0.685–1.124)	p = 0.301
More than 26	2.950 (1.768–4.921)	p = 0.000*	0.914 (0.606–1.376)	p = 0.665
**Marital status**				
Single	Reference	p = 0.001*	Reference	p = 0.456
Married	2.303 (1.417–3.744)	p = 0.001*	1.074 (0.750–1.538)	p = 0.697
Divorced/widowed	0.619 (0.227–1.687)	p = 0.349	0.609 (0.249–1.493)	p = 0.278
**Nationality**		p = 0.000*		p = 0.081
UAE National	Reference		Reference	
Resident Expatriate	0.423 (0.346–0.517)		0.831 (0.675–1.023)	
**Current educational level**		p = 0.610		p = 0.307
Undergraduate	Reference		Reference	
Postgraduate	0.891 (0.572–1.389)		0.824 (0.568–1.195)	
**College level**				
Freshmen	Reference	p = 0.062	Reference	p = 0.002*
Sophomore	0.986 (0.790–1.231)	p = 0.902	0.673 (0.536–0.845)	p = 0.001*
Senior	0.677 (0.474–0.968)	p = 0.032*	0.647 (0.460–0.910)	p = 0.012*
**COVID-19 vaccination status**		p = 0.815		p = 0.313
Vaccinated	Reference		Reference	
Unvaccinated	1.052 (0.686–1.615)		0.823 (0.563–1.202)	
**Previous COVID-19 infection**		p = 0.587		p = 0. 794
Yes	Reference		Reference	
No	0.939 (0.749–1.178)		0.971 (0.781–1.208)	

OR: Odds Ratio, CI: Confidence Interval. Dependent variable: perception scores, Independent variables: demographics.

* p value was based on binary logistic regression test at 5% level.

### Concerns and readiness for hybrid learning

The success of any educational system necessitates the harmonization of all of its elements such as instructors, students, syllabi, assessment, resources, and amenities [14]. Due to the instability and rapid shift of learning approaches amid the pandemic, students can experience greater stress and distraction [14]. Available literature reports show certain difficulties for both instructors and students during conducting online classes such as unfamiliarity with the online platform used, internet access limitations, and inadequate technological experience among others [33, 34]. Overall, most of the students were aware of the COVID-19 precautionary measures as shown in Table 4. Around a quarter of the students reported taking special preparations for hybrid learning (24.0%). Vaccination was the most popular preparation among students, followed by building strong hygiene habits and living away from home (62.0%, 36.4%, and 13.4% respectively). Around a third of the students expressed feeling uncomfortable when visiting the university campus while on hybrid learning (39.4%). When asked about the reasons, students reported fear of infection (25.2%), worries about family members (28.8%), and the belief that online learning is more flexible and easily accessible from any place (44.9%). Regarding the expected difficulties while in hybrid learning, more than half of the students reported a reduced level of focus (55.5%), confusion in the learning sitting (53.5%), followed by technical problems (39.6%).

**Table 4:** Students’ concerns and readiness for hybrid learning (n = 2056).

Variables	n	%
**Taken special preparations for hybrid learning**		
Yes	493	24.0
No	1563	76.0
**If yes, what are they** *		
Vaccination	1275	62.0
Living away from home	276	13.4
Build strong hygiene habits	748	36.4
Others	325	15.8
**I feel comfortable when I visit the university campus during hybrid learning**		
Yes	810	39.4
No	1246	60.6
**If not, why** *		
Fear of infection	519	25.2
Worries about family members	593	28.8
Not convinced with the vaccine	99	4.8
Online is more flexible and accessible from any place	923	44.9
Others	211	10.3
**Expected difficulties during hybrid learning**		
The level of focus in learning is very different between online learning and face-to-face learning	1142	55.5
Technical problems and difficulties	814	39.6
Confusion in the learning setting while moving between online and face-to-face learning	1100	53.5
Others	406	19.7

* As multiple responses were allowed, the total number of responses is greater than the number of surveyed participants, and the percentage of cases is displayed.

### Changes in university life during hybrid learning

**Fig 1:**
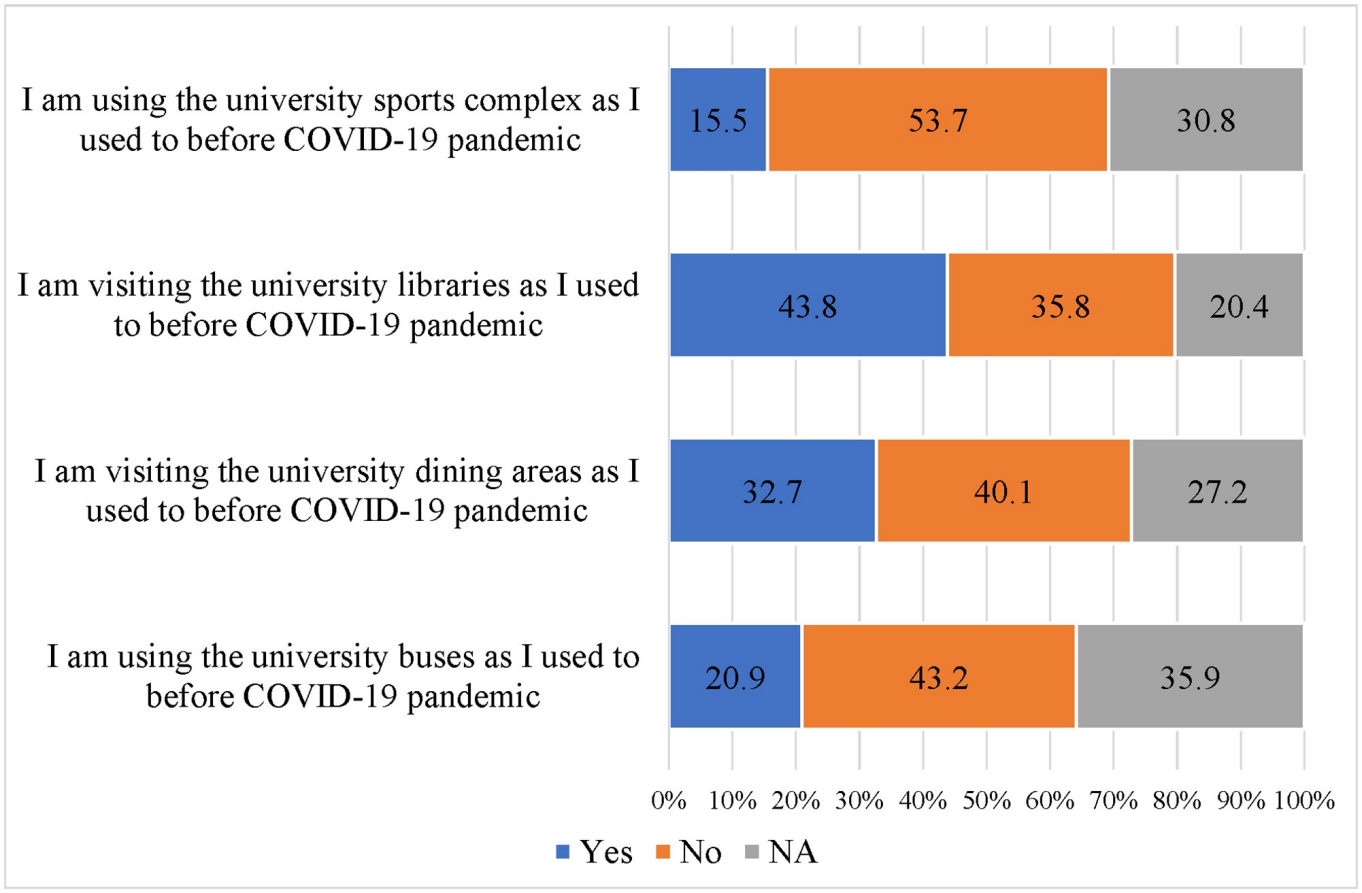
Changes in university life during hybrid learning.

Fig 1 shows the distribution of the students reporting changes in their university life after the implementation of hybrid learning. About 44% and 33% of the students reported no change in visiting the university libraries and dining areas as they used to before the COVID-19 pandemic respectively. A lesser percentage of students reported using the university buses and going to the university sports complex (21% and 16% respectively).

In efforts to reassure students, staff, faculty, and parents, several educational institutions in the UAE emphasized their commitment to implementing all required precautionary measures to ensure the health and safety of all, upon returning to face-to-face learning. These measures included periodic sterilization for all buildings and facilities while maintaining social distancing, use of face masks, and routine COVID-19 testing [35, 36]. A Spearman’s rank-order correlation was performed to investigate the relationship between students’ perceptions and four specific behaviors related to the use of university facilities during the hybrid learning mode as presented in Table 5. The results showed that as students’ perceptions of hybrid learning improved, their use of university buses (rs = 0.070, p = 0.002), dining areas (rs = 0.131, p < 0.001), libraries (rs = 0.154, p < 0.001), and sports complex (rs = 0.113, p < 0.001) tended to increase as well. However, the strength of these correlations was weak. It is important to note that correlation does not imply causation, and other factors not measured in this study may also influence students’ perceptions and behaviors. Nonetheless, the statistically significant correlations suggest that there is a low probability that these findings are due to chance alone.

**Table 5:** Correlation between perceptions and behaviors.

	Perceptions of hybrid learning
Behaviors	Correlation coefficient
I am using the university buses as I used to before the COVID-19 pandemic	0.070*
I am visiting the university dining areas as I used to before the COVID-19 pandemic	0.131*
I am visiting the university libraries as I used to before the COVID-19 pandemic	0.154*
I am using the university sports complex as I used to before the COVID-19 pandemic	0.113*

*Correlation is significant at the 0.01 level (2-tailed)

UNESCO has been keen on studying the effects of COVID-19 disruption on higher education teaching, student education, research, finances, and mobility in several regions. This yielded a report titled ‘Resuming or Reforming’, indicating that the swift return to traditional teaching after two years into the COVID-19 pandemic will not cause a profound transformation of the conventional face-to-face experience. Moreover, UNESCO called on institutions to create a more unbiased post-pandemic education atmosphere and enhance hybrid learning [37].

It is important to consider the strengths and limitations when interpreting the findings of this study. A major strength of this study was that it was the first in the UAE to analyze students’ perceptions of hybrid learning. The study provided key insights into students’ thoughts on the hybrid learning mode and their concerns and difficulties. Moreover, the guaranteed anonymity of the students may have provided them with more convenience and confidence in transparently reporting their opinions and behaviors. However, this study applies to students at the university of Sharjah at a specific period; that is at the early stages of the hybrid mode implementation. The results of the study therefore may underrepresent students’ perceptions throughout the whole duration of the implementation of the system. Moreover, the use of a self-reported survey may lead to respondent bias or misreporting of data. Also, the higher proportion of men to women and data collection within one university might impact the generalizability of the results. Incorporating multiple universities which adopted similar learning styles across the region, besides selecting the participants in a more randomized manner would increase the external validity of the study [38].

## Conclusions

This study offers insights into how university students were affected by the COVID-19 pandemic and an evaluation of their experience in applying online and hybrid learning at the University of Sharjah. Most students preferred online or on-campus over hybrid learning. Generally, students had positive perceptions of online and hybrid learning in terms of university support, while most students agreed they understand the course material better through on-campus learning than in distance learning. The main difficulties faced by the students in hybrid learning were motivation and discomfort when on campus and distraction due to mixed methods. Men, older students, and those who are married had better perceptions of online learning, while sophomore students had better perceptions of hybrid learning. The findings of the study call for future research on the efficiency of hybrid learning in the post-pandemic reality. Moreover, educational institutes need to observe the long-term effects of distance learning particularly for students who commenced started their educational journey during the pandemic and adapted to online and hybrid learning modes. Furthermore, obstacles and concerns should be considered for future planning to ensure the resilience and proactivity of the educational system.

## Supporting information

S1 Table Perceptions towards online and hybrid learning (n = 2056). (DOCX)
