# Supplementary material for: Comparison of students’ perceptions of online and hybrid learning modalities during the covid-19 pandemic: The case of the University of Sharjah
Source: PLoS One. 2023 Mar 28;18(3):e0283513. doi: 10.1371/journal.pone.0283513 (PMC10047520; doi:10.1371/journal.pone.0283513)
Supplement: S1 Table — (DOCX) [file pone.0283513.s001.docx]

**Table S1: Perceptions of online and hybrid learning (n=2056)**

| **Item** | **Positive** | **Negative** |
| --- | --- | --- |
| **Perceptions of online learning** | | |
| I got used to the online learning experience | 1327 (64.5%) | 729 (35.5%) |
| The university provided you with the needed technical support | 1354 (65.9%) | 702 (34.1%) |
| The university provided you with the needed personal support | 1118 (54.4%) | 938 (45.6%) |
| It was easy for me to communicate with my instructor in online classes | 1290 (62.7%) | 766 (37.3%) |
| It was easy for me to ask questions during online classes | 1325 (64.4%) | 731 (35.6%) |
| It was easy for me to engage with the professor in distance learning | 1159 (56.4%) | 897 (43.6%) |
| The instructor was available during office hours | 1581 (76.9%) | 475 (23.1%) |
| Explanations and lecturing in theoretical lectures are better in online classes | 1076 (52.3%) | 980 (47.7%) |
| Practical sessions are better in online classes | 881 (42.9%) | 1175 (57.1%) |
| Questions in online exams were easy to read and understand | 1293 (62.9%) | 763 (37.1%) |
| Online exams are fair | 1242 (60.4%) | 814 (39.6%) |
| The online exam format was better than the in-person exams | 1180 (57.4%) | 876 (42.6%) |
| Grading in online exams was fair and better than in in-person exams | 1055 (51.3%) | 1001 (48.7%) |
| Proctoring online exams were not effective | 446 (21.7%) | 1610 (78.3%) |
| It was easy to cheat in online exams | 329 (16.0%) | 1727 (84.0%) |
| **Perceptions of hybrid learning** | | |
| Instructions and guidelines are provided regularly | 1614 (78.5%) | 442 (21.5%) |
| The university provides us with excellent online support services | 1513 (73.6%) | 543 (26.4%) |
| The university provides students with excellent technical support | 1400 (68.1%) | 656 (31.9%) |
| The university provides us with excellent personal support | 1211 (58.9%) | 845 (41.1%) |
| The university provides us with financial aid and support | 846 (41.1%) | 1210 (58.9%) |
| I understand the course material better through on-campus learning | 1414 (68.8%) | 642 (31.2%) |
| I achieve better in hybrid classes compared to online classes | 919 (44.7%) | 1137 (55.3%) |
| I feel comfortable when visiting the university campus during hybrid learning | 810 (39.4%) | 1246 (60.6%) |
| I feel unmotivated to be in a hybrid learning system | 674 (32.8%) | 1382 (67.2%) |
| I am able to manage my time better in hybrid classes compared to online classes | 860 (41.8%) | 1196 (58.2%) |
| A hybrid learning system could be distracting due to mixed learning methods | 476 (23.2%) | 1580 (76.8%) |
